# Surgical load in major fractures — results of a survey on the optimal quantification and timing of surgery in polytraumatized patients

**DOI:** 10.1007/s00264-023-05828-4

**Published:** 2023-05-17

**Authors:** Felix Karl-Ludwig Klingebiel, Morgan Hasegawa, Oliver Strähle, Yannik Kalbas, Michel Teuben, Sascha Halvachizadeh, Yohei Kumabe, Hans-Christoph Pape, Roman Pfeifer, SICOT Trauma Research Group, Turki Bashir Al-Rouk, Bergita Ganse, Marc Hanschen, Ilir Hasani, Gleb Korobushkin, Jeannie McCaul, Joshua A. Parry, Mohamed Rashed, Jordan Saveski, Hemant Sharma, Mohammed Zarti, Boris A. Zelle

**Affiliations:** 1 Department of Traumatology, University Hospital Zurich, Rämistrasse 100, 8091 Zurich, Switzerland; 2 Department of Surgical Research, Harald Tscherne Laboratory for Orthopaedic and Trauma Research, Zurich University Hospital, Zurich, Switzerland; 3 Division of Orthopaedic Surgery, John A. Burns School of Medicine, University of Hawai’i, Honolulu, HI USA; 4 Department of Orthopaedic Surgery, Kobe University Graduate School of Medicine, Chuo-Ku, Kobe, 650-0017 Japan

**Keywords:** Polytrauma, Surgical treatment strategy, Safe definitive surgery, Surgical load, Damage control

## Abstract

**Purpose:**

It is known that the magnitude of surgery and timing of surgical procedures represents a crucial step of care in polytraumatized patients. In contrast, it is not clear which specific factors are most critical when evaluating the surgical load (physiologic burden to the patient incurred by surgical procedures). Additionally, there is a dearth of evidence for which body region and surgical procedures are associated with high surgical burden. The aim of this study was to identify key factors and quantify the surgical load for different types of fracture fixation in multiple anatomic regions.

**Methods:**

A standardized questionnaire was developed by experts from Société Internationale de Chirurgie Orthopédique et de Traumatologie (SICOT)-Trauma committee. Questions included relevance and composition of the surgical load, operational staging criteria, and stratification of operation procedures in different anatomic regions. Quantitative values according to a five-point Likert scale were chosen by the correspondents to determine the surgical load value based on their expertise. The surgical load for different surgical procedures in different body regions could be chosen in a range between “1,” defined as the surgical load equivalent to external (monolateral) fixator application, and “5,” defined as the maximal surgical load possible in that specific anatomic region.

**Results:**

This questionnaire was completed online by 196 trauma surgeons from 61 countries in between Jun 26, 2022, and July 16, 2022 that are members of SICOT. The surgical load (SL) overall was considered very important by 77.0% of correspondents and important by 20.9% correspondents. Intraoperative blood loss (43.2%) and soft tissue damage (29.6%) were chosen as the most significant factors by participating surgeons. The decision for staged procedures was dictated by involved body region (56.1%), followed by bleeding risk (18.9%) and fracture complexity (9.2%). Percutaneous or intramedullary procedures as well as fractures in distal anatomic regions, such as hands, ankles, and feet, were consistently ranked lower in their surgical load.

**Conclusion:**

This study demonstrates a consensus in the trauma community about the crucial relevance of the surgical load in polytrauma care. The surgical load is ranked higher with increased intraoperative bleeding and greater soft tissue damage/extent of surgical approach and depends relevantly on the anatomic region and kind of operative procedure. The experts especially consider anatomic regions and the risk of intraoperative bleeding as well as fracture complexity to guide staging protocols. Specialized guidance and teaching is required to assess both the patient’s physiological status and the estimated surgical load reliably in the preoperative decision-making and operative staging.

**Supplementary Information:**

The online version contains supplementary material available at 10.1007/s00264-023-05828-4.

## Introduction

Trauma remains one of the greatest burdens on human society, and treatment strategies for severely injured patients continue to be the subject of intense debate [1]. Proponents of early fracture fixation cite benefits in fixation of major fractures allowing faster mobilization and lower risk of thromboembolic complications and systemic infection [2]. However, in multiply injured patients, early invasive surgical procedures may impart a “second hit” phenomena, leading to further decompensation and increasing risk of complications [3]. The second hit phenomenon describes inflammatory, biochemical, and physiologic changes in patients due to surgery or post-traumatic clinical course (e.g., infections, thromboembolism) following a major trauma. The trauma itself is referred to as the “first hit” that disrupts the patient’s physiology in pulmonary, coagulatory, inflammatory, and further systemic pathways [4]. Since the human body can only compensate a certain amount of impairment before entering a critical state (e.g. sepsis, MODS), it is established to adjust the initial surgical treatment of polytrauma patients and therefore the second hit to the patient’s physiology [3]. This strategy is also called Damage Control Orthopedics. In addition to the timing of surgery, the extent of surgery is also important in clinical decision-making as the surgeon can select from a variety of surgical approaches, implants, and degrees of fracture stabilization. We propose that the quantifiable amount of physiologic effect imparted by surgery should be referred to as the “surgical load,” to weigh different procedures against each other.

There is limited scientific evidence on the effects of different surgical procedures on physiology and outcome. Still, most of the decision-making when considering the balance between patient’s physiologic status and additional load incurred from surgery is based on clinical experience and individual hospital standards. The aim of this study was to identify key factors and quantify the surgical load for different types of fracture fixation in multiple anatomic regions by performing an international survey in the scientific trauma community to assist guidance in operative decision-making and staging protocols.

## Methods

### Study design

The initial survey was developed by the Société Internationale de Chirurgie Orthopédique et de Traumatologie (SICOT) Trauma committee and other experts of polytrauma care. Pilot study: The survey was tested by experienced trauma surgeons (S.H, M.T R.P, H-C.P) and members of the SICOT trauma committee; annotations and suggestions were implemented. The survey was then disseminated amongst members of SICOT, and responses were collected after voluntary participation and submission of survey responses.

### Ethics approval statement

The survey was anonymous and voluntary. All participants agreed to the use of their provided data. The local ethic committee disclosed a general waiver for anonymous surveys.

### Survey

The questionnaire was offered between June 1 and August 30, 2022 consisting of twelve possible selections between four different categories (Electronic Supplementary Material Appendix).

- Sociodemographic data (gender, country, working experience, level of education and frequency of polytrauma treatment) (five questions)
- Relevance of the surgical load in polytrauma patients and consideration in staged surgery procedures (two questions; Likert scale)
- Prioritization of multiple parameters in terms of assessing the surgical load and sequencing of operations (two questions; priority selection)
- Quantification of the surgical load by different surgical procedures in three anatomic regions (upper extremity, lower extremity, trunk) (three questions; Likert Scale)

The survey function in Google forms (Google LLC, “Assessment of surgical load”, accessed Jul 16, 2022, https://forms.gle/Z4pgR3PiqM5Ef7hu7) was used by which anonymity was guaranteed to all participants. The online survey was distributed to the members of SICOT. One reminder was sent after four weeks to members of SICOT.

### Statistical analysis

Categorical variables are shown as count and percentages, continuous variables as mean or median with standard deviation. Groups of continuous variables were compared using the student’s *t* test. Graphics were created utilizing the R-package ggplot (R Core Team (2019), R Foundation for Statistical Computing, Vienna, Austria (https://www.R-project.org) [5]. A *p*-value < 0.05 was considered as moderate significance, < 0.01 as strong significance, and < 0.001 as very strong significance according to Fisher [6]. Statistical analysis was performed using R ((R Core Team (2019), R Foundation for Statistical Computing, Vienna, Austria (https://www.R-project.org)).

## Results

### Participant’s demographics

A total of 196 participants from 61 different countries completed the questionnaire. Most participants were attending surgeons (*n* = 103, 52.6%) or department heads (*n* = 71, 36.2%). Surgeons from Asia were most represented (*n* = 109, 55.6%), followed by Europe (*n* = 37, 18.9%) and Africa (*n* = 27, 13.8%). A majority of participants are male (*n* = 186, 94.9%), have a median of 13 years of working experience in trauma care, and treat a median of 5.5 polytrauma patients per month (Table 1, Electronic Supplementary Material Appendix).

**Table 1:** Demographics of participants

Characteristics	Participants (*N* = 196)
Sex, *n* (%)	
Male	186 (94.9)
Female	10 (5.1)
Level of education, *n* (%)	
Head of Department	71 (36.2)
Attending	103 (52.6)
Resident	21 (10.7)
Intern	1 (0.5)
Years of working experience, median (mean ± SD)	13 (16.22 ± 10.88)
Frequency of polytraumatized patients per month, median (mean ± SD)	5.5 (17.74 ± 45.54)
Continents of origin, *n* (%)	
Africa	27 (13.8)
Asia	109 (55.6)
Australia	4 (2.0)
Europe	37 (18.9)
North America	7 (3.6)
South America	10 (5.1)
N/A	2 (1.0)
Countries of origin (Table 7), *n*	61

### Relevance of the surgical load/impact on staging

**Fig. 1:**
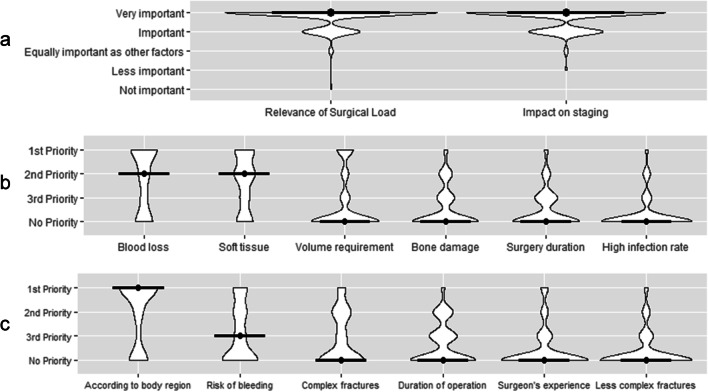
**a** Relevance of the surgical load. **b** Parameters for the surgical load. **c** Staging criteria

One hundred fifty-one participants (77%) rated the assessment of the surgical load on polytraumatized patients as very important, and 41 participants (20.9%) rated surgical load as important. In addition, 192 trauma surgeons (97.9%) rate the adjustment of the surgical load in the surgical staging procedures according to the patient’s physiology as important (26.5%) or very important (71.4%) (Fig. 1a; Table 2).

**Table 2:** Relevance of surgical load

Question	*N* (%)
A	
How relevant is the assessment of the surgical load on a patient after polytrauma?	196 (100)
Very important	151 (77.0)
Important	41 (20.9)
I don ‘t know/Equally important as other factors	3 (1.5)
Less important	0 (0)
Not important	1 ( 0.5)
B	
Is it useful to adjust the surgical load of secondary and following surgeries to a patient’s physiology after polytrauma	196 (100)
Very important	140 (71.4)
Important	52 (26.5)
I don ‘t know/ Equally important as other factors	3 ( 1.5)
Less important	1 ( 0.5)
Not important	0 (0.0)

### Assessment of the surgical load/relevance of parameters

Eighty-five participants (43.4%) chose intraoperative blood loss as the most important parameter for assessing the surgical load followed by severity of intraoperative soft tissue damage/extent of surgical approach (*n* = 58, 29.6%) and volume requirements/need for vasopressors (*n* = 31, 15.8%). When asked to select the second most important factor, severity of intraoperative soft tissue damage/extent of surgical approach was chosen (*n* = 62, 31.6%) followed by intraoperative blood loss (*n* = 34, 17.3%) and severity of bone damage (*n* = 26, 13.3%). As third priority, duration of surgery (*n* = 44, 22.4%) was most often selected, while severity of bone damage (*n* = 35, 17.9%) and severity of soft tissue damage/extent of surgical approach (*n* = 27, 13.8%) followed. When displayed in a violin plot with calculated median of response-count (Fig. 1b), the condition of blood loss ranks highest in terms of prioritization followed closely by soft tissue damage/extent of surgical approach. Less prioritized but still identified as factors for assessing surgical load were the remaining parameters in following order: volume requirement/need for vasopressors, bone damage, duration of surgery, and high infection rate (Table 3).

**Table 3:** Which parameters are relevant for the assessment of the surgical load in secondary and following surgeries?

Assessment of the surgical load	*N* (%)
First priority	
Intraoperative blood loss	85 (43.4)
Severity of intraoperative soft tissue damage/extensive surgical approach	58 (29.6)
Volume requirements/ need for vasopressors	31 (15.8)
Severity of bone damage	7 ( 3.6)
Duration of surgery	6 ( 3.1)
High infection rate	4 ( 2.0)
N/A	5 ( 2.6)
Second priority	
Severity of intraoperative soft tissue damage/extensive surgical approach	62 (31.6)
Intraoperative blood loss	34 (17.3)
Severity of bone damage	26 (13.3)
Duration of surgery	16 ( 8.2)
Volume requirements/ need for vasopressors	14 ( 7.1)
High infection rate	13 ( 6.6)
N/A	31 (15.8)
Third priority	
Duration of surgery	44 (22.4)
Severity of bone damage	35 (17.9)
Severity of intraoperative soft tissue damage/extensive surgical approach	27 (13.8)
Intraoperative blood loss	21 (10.7)
High infection rate	18 ( 9.2)
Volume requirements/ need for vasopressors	18 ( 9.2)
N/A	33 (16.8)

### Sequencing of surgery

Most participants (*n* = 110, 56.1%) stated their sequence of staged procedures was dictated by body region and risk of bleeding (*n* = 37, 18.9%). As second priority in decision making for sequence of staged surgical procedures, addressing complex fractures (23.5%) followed closely by bleeding risk (*n* = 35, 17.9%) and duration of the operation (*n* = 34, 17.3%) was selected by participating surgeons. As third priority, duration of procedure was most often selected (*n* = 47, 24%). When displayed in a violin plot and median of response-count is calculated, decisions involved with staging procedure is most often dictated in accordance to body region, followed by the risk of bleeding and complexity of fractures (Fig. 1c). The duration of procedures was also chosen as a relevant consideration, but appeared to be considered as a less prominent factor. Of lesser consideration and prominence in the decision-making matrix appeared to be surgeon experience and fractures of minor complexity (Table 4).

**Table 4:** How do you sequence secondary and following surgeries in cardiopulmonary compensated multiply injured patients?

Sequencing of surgeries	*N* (%)
First priority	
According to body region (i.e. trunk first, long bones second etc.)	110 (56.1)
Risk of bleeding	37 (18.9)
Complex fractures first	18 ( 9.2)
Experience of the surgeon	12 ( 6.1)
Less complex fractures first	6 ( 3.1)
Duration of operation	5 ( 2.6)
N/A	8 ( 4.1)
Second priority	
Complex fractures first	46 (23.5)
Risk of bleeding	35 (17.9)
Duration of operation	34 (17.3)
According to body region (i.e. trunk first, long bones second etc.)	24 (12.2)
Less complex fractures first	13 ( 6.6)
Experience of the surgeon	12 ( 6.1)
N/A	32 (16.3)
Third priority	
Duration of operation	47 (24.0)
Risk of bleeding	29 (14.8)
Experience of the surgeon	26 (13.3)
Complex fractures first	24 (12.2)
Less complex fractures first	24 (12.2)
According to body region (i.e. trunk first, long bones second etc.)	9 ( 4.6)
N/A	37 (18.9)

### Severity of the surgical load dealt by different operational procedures

**Fig. 2:**
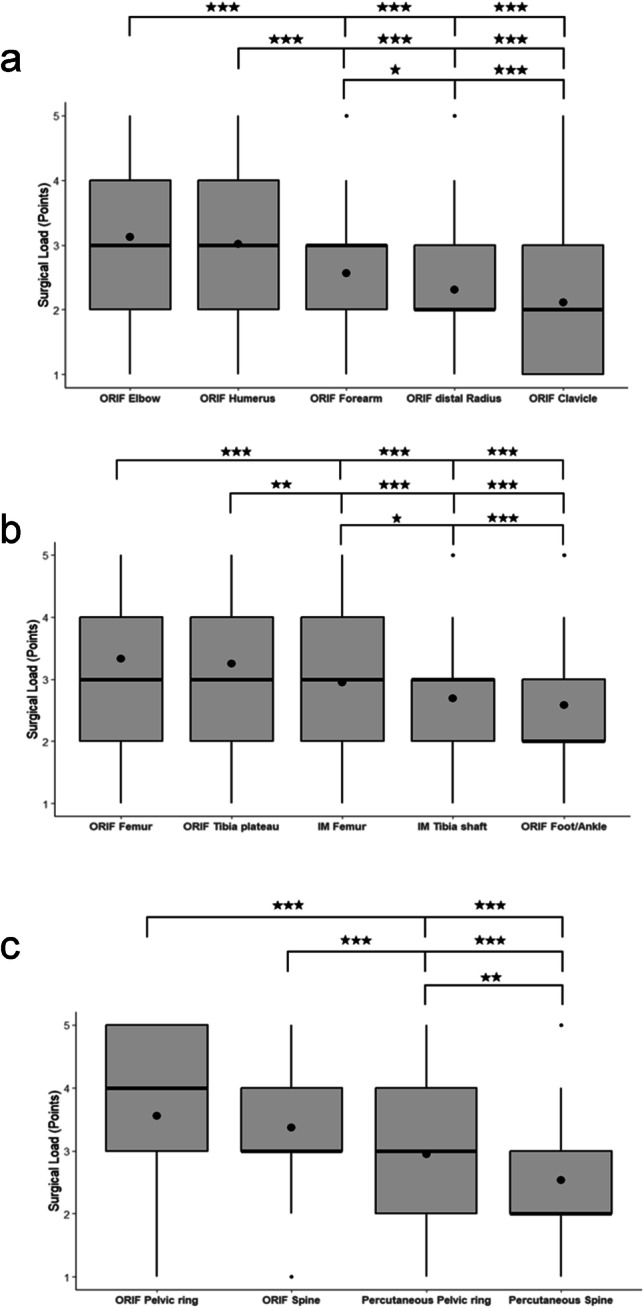
Quantification of the surgical load. **a** Upper extremity. **b** Lower extremity. **c** Trunk

Regarding the upper extremity, open reduction and internal fixation (ORIF) of the elbow (mean: 3.12 ± 1.3 points) and the humerus (mean: 3.02 ± 1.20 points) was ranked significantly higher (p < 0.0001) than ORIF of the forearm (mean: 2.56 ± 0.92 points), distal radius (mean: 2.30 ± 1.04 points), and clavicle (mean 2.11 ± 1.14 points). ORIF of the forearm was ranked moderately higher (p = 0.02) than ORIF of the distal radius and clavicle (*p* < 0.0001). ORIF of the distal radius and clavicle was comparable to each other (*p* = 0.012). (Fig. 2a; Table 5).

Regarding the lower extremities, ORIF of the femur (mean: 3.34 ± 1.30 points) and tibial plateau (mean: 3.25 ± 1.19 points) were evaluated as significantly higher in their surgical load than intramedullary nailing of the femur (mean: 2.95 ± 1.32 points; *p* = 0.07, resp. *p* = 0.027), tibia (mean 2.69 ± 1.10, *p* < 0.0001), as well as ORIF of the foot and ankle (mean 2.58 ± 1.12 points). Intramedullary nailing of the tibia and ORIF of the foot/ankle were rated as comparable (*p* = 0.37) in terms of their surgical load (Fig. 2b; Table 5).

Considering the trunk, open reduction and internal fixation of the pelvic ring (mean: 3.56 ± 1.43 points) and open spine procedures (mean: 3.37 ± 1.13 points) were ranked significantly higher (*p* < 0.0001) than percutaneous procedures in the same region (pelvis, mean: 2.95 ± 1.44 points; spine: mean: 2.54 ± 1.11 points). Percutaneous stabilization of the pelvic ring was chosen as imparting a higher surgical load than percutaneous procedures of the spine (*p* = 0.004) (Fig. 2c; Table 5).

**Table 5:** Quantification of the surgical load

Operation	Surgical Load (Mean ± SD)
Upper extremity	
Open reduction internal fixation of the elbow	3.12 ± 1.30
Open reduction internal fixation of the humerus	3.02 ± 1.20
Open reduction internal fixation of the forearm	2.56 ± 0.92
Open reduction internal fixation of the distal Radius	2.30 ± 1.04
Open reduction internal fixation of the clavicle	2.11 ± 1.14
Lower extremity	
Open reduction internal fixation of the femur	3.34 ± 1.30
Open reduction internal fixation of the tibia plateau	3.25 ± 1.19
Intramedullary nailing of the femur shaft	2.95 ± 1.32
Intramedullary nailing of the tibia shaft	2.69 ± 1.10
Open reduction internal fixation of the foot/ankle	2.58 ± 1.12
Trunk	
Open reduction internal fixation of the pelvic ring	3.56 ± 1.43
Open spondylodesis of the spine	3.37 ± 1.13
Anterior and posterior percutaneous pelvic stabilization of an unstable pelvic ring	2.95 ± 1.44
Percutaneous spine fixation	2.54 ± 1.11

## Discussion

Consideration of surgery’s physiologic burden contributes to the decision making concerning surgical timing or staging in a polytraumatized patient. We aimed to evaluate how an international body of orthopedic trauma surgeons evaluates factors concerning surgical load and stratification of surgical procedures. Our comprehensive survey, in which a large number of orthopedic surgeons (*n* = 196) participated, yielded the following main findings:

1. Assessment of the surgical load in polytrauma patients was rated as (very) important by almost all participants.
2. The main contributors to the surgical load are the extent of surgical exposure (soft tissue damage) and the risk of bleeding.
3. The majority of orthopedic surgeons stage secondary procedures based on anatomic region, bleeding risk, and/or complexity of fracture fixation.

### Relevance of the surgical load/impact on staging

Our survey found that the majority of participating orthopedic surgeons consider the assessment of the surgical load in the treatment of polytraumatized patients important or very important. Yet, even with a general consensus amongst an international constituency, there remain no defined standards for quantifying surgical load. Several studies have attempted to measure the postoperative release of cytokines and other inflammatory mediators such as interleukin-6 and IL-8 and have found varying effects of surgery on the inflammatory response [7, 8]. Analysis of the systemic inflammatory response after limb or trunk fracture surgery showed the proinflammatory response was higher in patients after pelvic surgery than in those patients undergoing spinal fracture instrumentation [9]. Furthermore, cytokine response was also associated with the degree of blood loss [10]. When evaluating how these makers related to surgery, the authors found the amount of markers released was associated with the extent of surgery, rather than the duration of surgery. Evaluation of these markers in severe polytraumatized patients is challenging, as systemic release of inflammatory mediators after initial trauma is at such high levels that isolating subsequent changes in these markers after surgery can be difficult [11]. These results match with the findings of our study as soft tissue damage by the surgical approach and bleeding are rated as most important in the composition of the surgical load.

Moreover, it has also been shown that surgical interventions and procedures can lead to local tissue damage and activation of a local inflammatory response and recruitment of immune cells [11]. Extensive surgical damage is associated with local tissue devascularization and decreased tissue perfusion, leading to increased risk of infection and subsequent wound healing [12]. Concerns for local blood loss is also present, considering muscle is a well-perfused tissue, allowing hemorrhage to occur rapidly, leading to expanded areas of tissue necrosis in initially unaffected regions [13].

The local signaling cascade after surgical insult also has the potential to send cytokines, cell signaling mediators, and other mediators into systemic circulation, inducing a systemic response [14]. These changes and downstream effects after surgical procedures must be considered, when determining if the physiologic burden of surgery can be tolerated by a patient with a pre-existing physiologic compromise due to surgery. Orthopaedic surgeons have been advised to select procedures to ensure the biologic or physiologic cost does not exceed the patient’s capacity to maintain a state of physiologic stability. Failure to do so may result in additional physiologic insult to an already compromised patient, potentially leading to adverse outcome and additional complications [11].

### Relevance of parameters

Intraoperative bleeding is one of the most common complications in surgery. In most cases, severe blood loss is associated with vascular injury, leading to increased mortality, morbidity, and intensive care treatment [15]. Prolonged haemorrhage can exacerbate dilution effects on blood clotting, induce hypothermia and acidosis, further exacerbating the vicious cycle of shock [16]. Early diagnosis and treatment of coagulopathy has been shown to facilitate earlier treatment of those with musculoskeletal injuries [17]. In polytrauma patients with brain injury, it is particularly important to consider blood loss because hypovolemia in these patients is associated with secondary brain injury and resultant hypotension and hypoxia [18]. Percutaneous procedures of the pelvis and spine have gained refinement and increased use over the past few decades [19, 20]. Less invasive or percutaneous procedures may allow earlier, or emergent, stabilization of pelvis and spine fractures, due to the decreased physiologic burden [21–23]. This matches with our results, as percutaneous interventions are rated lower in their surgical load and therefore are expected to deliver a smaller impact on the patient’s physiology.

### Sequencing of surgery

Multiple strategies were developed to treat polytraumatized patients according to their injury pattern and physiology [24]. Those strategies need to be adapted individually to every patient to manage the balance between early survival after trauma and early reconstruction with restoring mobility [25]. The concept of staged and delayed surgery for polytrauma patients has evolved over the past few decades, particularly with regard to adjusting the surgical burden. The early total care concept (ETC), which often exceeded the compensable surgical load [26, 27], has been followed by the damage control orthopaedics (DCO). This strategy focused on using external fixation techniques to keep the surgical load as low as possible [28, 29]. This strategy has other disadvantages, which are associated with longer immobilization, increased infection rates, extended in-hospital stay, and higher costs. Those two strategies have been merged to a more dynamic concept called safe definitive surgery (SDS). This allows the surgeon to react and adjust the surgical load to the patient’s physiology dynamically by switching between temporary fixation and definitive osteosynthetic if reasonable [30]. Nowadays, we can assess the patient’s status based on multiple parameters and distinguish in between “stable,” “borderline,” “unstable,” and “in extremis” patients [3, 31] and chose the operational procedures with the individually compensable surgical load. Recently, it has been advocated that safe definitive surgery (SDS) is essential in avoiding complications [36]. If the principles of the SDS approach are followed, it appears that for secondary surgery, a window of opportunity and the avoidance of surgeries at days two to five post injury is no longer required but can be performed as soon as the physiological parameters have normalized [24].

It is proposed to define the status of the patient by using multiple systemic parameters and reassess them frequently. Critical analysis of these various pathophysiologic cascades, including the acid–base system, coagulation, hypothermia, and tissue damage, may allow improved predictions of early complications and outcomes [32]. Another benefit of stratifying patients to treatment strategies based on physiologic status is an enhanced ability to plan surgical strategy and fracture fixation goals, understanding the preoperative risk of further physiologic insult from any sort of operative procedure [31].

In the meantime, we also gained knowledge not only in the field “when to operate” but also “what to operate (first).” For instance, a special focus needs to be drawn to major fractures which may fulfill a combination of the following characteristics: (1) has relevant effect on the patient’s physiology, (2) should be immediately addressed, (3) determines the clinical course and decision making, (4) is associated with relevant complications [34]. Taking the surgical load in regard while addressing this kind of fracture and design an individual treatment plan depending on the injury pattern may be beneficial for the patient as it eases the decision making for a staged procedure and operational sequencing.

There is not much literature on the sequence of surgical procedures in polytrauma patients. In common practice, surgeons dictate their order of operations mainly by anatomic regions of injury, with severe injuries to the trunk (pelvis and spine) taking precedence. This prioritization was also shown in our findings which agree with recent insights in the understanding of major fractures whereas fractures of the pelvis and spine have shifted into the focus of surgical decision making [37]. Interestingly, however, in our study, it was also suggested bleeding risk or complexity of fracture fixation be highly considered. Future studies should continue to explore objective data to define surgical load and stratify patients for optimal surgical timing and procedures. Identifying objective ways to measure a patient’s physiologic reserve to withstand surgical load and insult may lead to more standardized methods of evaluating these polytraumatized patients with regard to surgical timing and planning.

### Severity of the surgical load

Although minimally invasive procedures can logically be expected to have less impact on the patient physiology, it is critical to be able to distinguish between surgical exposure in different regions and alternative fixation strategies. This could allow the surgeon to perform an operation with less surgical load as part of the staged protocol and to weigh different operations against each other. This requires some quantification of surgical load, which our study reports. Definitive fixation should be performed if the patient’s condition permits, and the effects of the specific surgery can be compensated. As our study shows, the expected burden depends strongly on the anatomic region and the type of surgical strategy and must be determined individually for each patient. Surgery on the trunk (e.g., pelvis and spine) and fractures on long bones (e.g., humerus and femur) place more stress on the patient than osteosyntheses on distal regions (e.g., feet/ankles and hand). This certainly correlates with the finding of our study that blood loss and soft tissue damage are the most important parameters of surgical load, as these areas carry more soft tissue and are more perfused.

Based on the opinion of the experts in this study, future studies may also explore methods to confirm the general consensus that percutaneous procedures represent a lower surgical burden, which may justify a strategy of maximizing percutaneous procedures rather than multiple open procedures. Currently, however, the understanding of surgical burden in polytrauma patients is incomplete. We therefore hypothesize that the sequence of minimally invasive procedures with less surgical burden is safer for the patient than the combination of multiple complex surgical procedures with high risk of bleeding and soft tissue injury.

International collaboration in large societies such as SICOT is essential to pool the knowledge of experts and achieve an international consensus in polytrauma care that also incorporates new technical advances such as robotic or navigated procedures into standards of care. Specialized training in the treatment and assessment of polytrauma patients is required to reliably assess the patient’s condition and select the appropriate treatment algorithm based on the estimated surgical burden, adapted to the patient’s physiology.

## Conclusion

This study demonstrates a consensus in the trauma community about the crucial relevance of the surgical load in polytrauma care. The surgical load is ranked higher with increased intraoperative bleeding and greater soft tissue damage/extent of surgical approach and depends relevantly on the anatomic region and kind of operative procedure. The experts especially consider anatomic regions and the risk of intraoperative bleeding as well as fracture complexity to guide staging protocols. Specialized guidance and teaching is required to assess both the patient’s physiological status and the estimated surgical load reliably in the preoperative decision-making and operative staging.

### Study limitations

This expert opinion survey (evidence level IV) is limited to a certain degree. The questionnaire was provided to the entire SICOT society, but the demographic information of the ones not participating could not be retrieved. Therefore, it can only be assumed that the participating cohort is representative for the trauma society. Participants could mostly only choose from specified parameters/options that were defined as most relevant by former studies.

## Supplementary Information

Below is the link to the electronic supplementary material.

Supplementary file1 (DOCX 17 KB)

## Data Availability

Data, (further) materials, and code can be requested individually from the author team. The team of authors reserves the right to evaluate and decide individually to hand out the requested data/materials/code.

## Abbreviations

ETC: Early total care
DCO: Damage control orthopedics
ICU: Intensive care unit
IL: Interleukin
IM: Intramedullar(y)
MODS: Multiple organ dysfunction syndrome
ORIF: Open reduction internal fixation
SDS: Safe definitive surgery
SICOT: Société Internationale de Chirurgie Orthopédique et de Traumatologie (International Society of Orthopaedic Surgery and Traumatology)
