# Supplementary material for: Surgical load in major fractures — results of a survey on the optimal quantification and timing of surgery in polytraumatized patients
Source: Int Orthop. 2023 May 17;47(7):1677–87. doi: 10.1007/s00264-023-05828-4 (PMC10267260; doi:10.1007/s00264-023-05828-4)
Supplement: Supplementary file 1 — Supplementary file1 (DOCX 17 KB) [file 264_2023_5828_MOESM1_ESM.docx]

**Appendix**

**A1**

**Survey Google forms “Assessment of surgical load”**

1. Gender
   1. Male
   2. Female
2. Level of Education
   1. Intern
   2. Resident
   3. Attending
   4. Head of department
3. Years of working experience
   1. [Free to fill]
4. Frequency of treatment of polytraumatized patients per month
   1. [Free to fill]
5. Country of the current employment
   1. [Free to fill]

Assessment of Surgical Load

1. How relevant is the assessment of the surgical load (surgical hit) on a patient after polytrauma?
   1. Very important
   2. Important
   3. I don’t know/Equally important as other factors
   4. Less important
   5. Not important
2. Is it useful to adjust the surgical hit of secondary and following surgeries to a patient’s physiology after polytrauma?
   1. Very important
   2. Important
   3. I don’t know/Equally important as other factors
   4. Less important
   5. Not important/this does not play a role in my consideration
3. Which parameters are relevant for the assessment of the surgical hit in secondary and following surgeries?
   1. Intraoperative blood loss
   2. Severity of intraoperative soft tissue damage/ extensive surgical approach
   3. Severity of bone damage
   4. High infection rate
   5. Duration of surgery
   6. Volume requirements/ need for vasopressors
4. How do you sequence secondary and following surgeries in cardiopulmonary compensated multiply injured patients?
   1. According to body region (i.e. trunk first, long bones second etc.)
   2. Less complex fractures first
   3. Complex fractures first
   4. Risk of bleeding
   5. Duration of operation
   6. Experience of the surgeon

Surgical load analysis should consider simple fracture morphology without considering systemic factors and injury patterns

1. How severe do you estimate the surgical load in the following operative procedures of the upper extremity in simple extra-articular fractures? [From 1 (comparable to an External Fixation) – 5 (highest load for upper extremities)]
   1. Open reduction internal fixation of the clavicle
   2. Open reduction internal fixation of the humerus
   3. Open reduction internal fixation of the elbow
   4. Open reduction internal fixation of the forearm
   5. Open reduction internal fixation of the dist. radius
2. How severe do you estimate the surgical load in the following operative procedures of the lower extremity in simple extra-articular fractures? [From 1 (comparable to an External Fixation) – 5 (highest load for lower extremities)]
   1. Intramedullary nailing of the femur shaft
   2. Open reduction internal fixation for the femur
   3. Open reduction internal fixation of the tibial plateau
   4. Intramedullary nailing of the tibia shaft
   5. Open reduction internal fixation of the foot/ankle
3. How severe do you estimate the surgical load in the following operative procedures of the trunk?[From 1 (comparable to an External Fixation) – 5 (highest load for the trunk)]
   1. Percutaneous spine fixation
   2. Open spondolydesis of the spine
   3. Anterior and posterior percutaneous pelvic stabilization of an unstable pelvic ring
   4. Open reduction internal fixation of pelvic ring injuries
4. Personal commentary on the subject (optional)

**A2**

**Participating countries**

| **Country of the current employment** | **Participants, n (%)** |
| --- | --- |
| Algeria | 1 (0.5) |
| Australia | 4 (2) |
| Austria | 1 (0.5) |
| Bahrain | 1 (0.5) |
| Bangladesh | 2 (1) |
| Belgium | 1 (0.5) |
| Brazil | 4 (2) |
| Bulgaria | 1 (0.5) |
| Cameroon | 1 (0.5) |
| Canada | 2 (1) |
| Chile | 1 (0.5) |
| Colombia | 1 (0.5) |
| Cuba | 1 (0.5) |
| Denmark | 1 (0.5) |
| Egypt | 7 (3.6) |
| Ethiopia | 3 (1.5) |
| France | 3 (1.5) |
| Germany | 3 (1.5) |
| Greece | 3 (1.5) |
| Haiti | 1 (0.5) |
| Hong Kong | 2 (1) |
| Hungary | 2 (1) |
| India | 57 (29.1) |
| Iraq | 4 (2) |
| Israel | 2 (1) |
| Italy | 2 (1) |
| Japan | 2 (1) |
| Jordan | 1 (0.5) |
| Kazakhstan | 1 (0.5) |
| Kenya | 2 (1) |
| Libya | 3 (1.5) |
| Malaysia | 7 (3.6) |
| Mexico | 1 (0.5) |
| Nepal | 2 (1) |
| Netherlands | 1 (0.5) |
| Nigeria | 5 (2.6) |
| North Macedonia | 1 (0.5) |
| Oman | 1 (0.5) |
| Pakistan | 7 (3.6) |
| Panama | 2 (1) |
| Philippines | 7 (3.6) |
| Poland | 1 (0.5) |
| Qatar | 1 (0.5) |
| Romania | 1 (0.5) |
| Russia | 3 (1.5) |
| Saudi Arabia | 3 (1.5) |
| Serbia | 1 (0.5) |
| Singapore | 1 (0.5) |
| South Africa | 1 (0.5) |
| Spain | 1 (0.5) |
| Switzerland | 8 (4.1) |
| Syria | 1 (0.5) |
| Tanzania | 2 (1) |
| Thailand | 1 (0.5) |
| Tunisia | 1 (0.5) |
| Turkey | 2 (1) |
| Ukraine | 3 (1.5) |
| United Kingdom | 4 (2) |
| USA | 1 (0.5) |
| Venezuela | 3 (1.5) |
| Yemen | 1 (0.5) |
| N/A | 2 (1) |
